# Added value of sodium MRI in multiparametric MRI for WHO grade II astrocytoma surveillance during “watchful waiting”: initial experience

**DOI:** 10.1007/s00117-025-01531-0

**Published:** 2025-11-12

**Authors:** Daniel Cantré, Ioan Gemescu, Lars Gerigk, Armin M. Nagel, Marco Essig, Sönke Langner, Marc-André Weber

**Affiliations:** 1 Institute of Diagnostic and Interventional Radiology, Pediatric Radiology and Neuroradiology, Rostock University Medical Center, Ernst-Heydemann-Str. 6, 18057 Rostock, Germany; 2 Department of Diagnostic and Interventional Radiology, Hufeland Klinikum, Bad Langensalza, Germany; 3 Division of Medical Physics in Radiology, German Cancer Research Center (DKFZ), Heidelberg, Germany; 4 Institute of Radiology, University Hospital Erlangen, Friedrich-Alexander-Universität Erlangen-Nürnberg (FAU), Erlangen, Germany; 5 Department of Radiology, University of Manitoba, Winnipeg, Canada

**Keywords:** MRI in oncology, Brain tumor, Low grade astrocytoma, 3D radial gradient echo projection imaging sequence, DSC perfusion MRI, MRT in der Onkologie, Hirntumor, Niedriggradiges Astrozytom, 3D Radial Gradient Echo Projection Imaging Sequenz, DSC-Perfusions-MRT

## Abstract

**Background:**

Unresectable WHO grade II astrocytomas require continuous imaging surveillance. To evaluate whether sodium MRI (^23^Na-MRI) adds diagnostic value to multiparametric MRI and helps predict progressive disease (PD), patients monitored under a “watchful waiting” strategy were repeatedly examined.

**Materials and methods:**

Overall, 18 patients with biopsy-proven WHO grade II astrocytoma (10 female, mean age 42 ± 15 years) were prospectively included after baseline imaging. The imaging protocol comprised morphological MRI (T2 TSE, T2 FLAIR, pre- and post-contrast T1 SE), DSC perfusion MRI (*n* = 17), and ^23^Na-MRI (*n* = 9) at 3 T. At baseline, evaluable ^23^Na-MRI was available for six patients. The Response Assessment in Neuro-Oncology criteria were used to define PD. Semiquantitative ROI analysis was performed on DSC- and ^23^Na-MRI. Data were analyzed using the Cox regression model.

**Results:**

In 14 patients (78%), PD was found after a mean of 420 ± 354 days. For the endpoint time to progression, univariate Cox regression revealed a hazard ratio (HR) of 1.39 for relative regional cerebral blood volume (rrCBV) in the tumor at baseline, and an HR of 1.29 for relative regional cerebral blood flow (rrCBF) at baseline. The ^23^Na signal in tumor tissue at baseline, normalized to sodium phantoms, revealed an HR of 0.91.

**Conclusion:**

Elevation of rrCBV and rrCBF in the tumor indicates poor prognosis, in line with the literature. ^23^Na-MRI can be used for WHO grade II astrocytoma surveillance. In some treatment-naïve WHO grade II astrocytomas, an initially high sodium signal seems to be prognostically favorable, contrary to the literature on ^23^Na-MRI in postoperative aftercare. However, due to the small cohort size with evaluable ^23^Na-MRI at baseline, evidence is limited. In future, ^23^Na-MRI may help selecting patients for a “watchful waiting” strategy.

## Abstract (de)

**Hintergrund:**

Nichtresektable WHO Grad II Astrozytome erfordern eine kontinuierliche bildgebende Überwachung („Watchful-Waiting-Strategie“). Um zu ermitteln, ob die Natrium-Magnetresonanztomographie (^23^Na-MRT) den diagnostischen Wert der multiparametrischen MRT erhöht und dazu beiträgt, einen Tumorprogress vorherzusagen, wurden Patienten im Rahmen einer „Watchful-Waiting-Strategie“ wiederholt untersucht.

**Material und Methoden:**

Insgesamt konnten 18 Patienten mit bioptisch gesichertem WHO Grad II Astrozytom (10 Frauen, Durchschnittsalter 42 ± 15 Jahre) nach der bildgebenden Ausgangsuntersuchung prospektiv in die Studie eingeschlossen werden. Zum Untersuchungsprotokoll gehörten die morphologische MRT (T2-TSE, T2-FLAIR, Prä- und Postkontrast T1-SE), dynamische suszeptibilitätsgewichtete (DSC-)Perfusions-MRT (*n* = 17) und die ^23^Na-MRT (*n* = 9) bei 3 T. Am Beginn der Studie waren auswertbare ^23^Na-MRT-Daten für 6 Patienten verfügbar. Ein Tumorprogress wurde gemäß der „Response Assessment in Neuro-Oncology“-Kriterien bestimmt. Außerdem wurde eine semiquantitative Region-of-Interest(ROI)-Analyse der DSC- und ^23^Na-MRT durchgeführt. Die Daten wurden unter Verwendung des Cox-Regressions-Modells ausgewertet.

**Ergebnisse:**

Bei 14 Patienten (78%) wurde im Mittel nach 420 ± 354 Tagen ein Tumorprogress festgestellt. In Bezug auf den Endpunkt der Dauer bis zur Progression ergab die univariate Cox-Regressionsanalyse eine Hazard Ratio (HR) von 1,39 für das relative regionale zerebrale Blutvolumen (rrCBV) im Tumor zu Studienbeginn und eine HR von 1,29 für den relativen regionalen zerebralen Blutfluss (rrCBF) zu Studienbeginn. Für das ^23^Na-Signal im Tumorgewebe zu Studienbeginn, normiert auf Natriumphantome, zeigte sich eine HR von 0,91.

**Schlussfolgerung:**

Eine Erhöhung des rrCBV und rrCBF im Tumor ist – in Übereinstimmung mit der Literatur – ein Hinweis auf eine schlechte Prognose. Die ^23^Na-MRT kann während einer „Watchful-Waiting-Strategie“ bei WHO Grad II Astrozytomen im Rahmen der multimodalen MRT-Bildgebung eingesetzt werden. Bei einigen therapie-naiven Astrozytomen vom WHO Grad II scheint – im Gegensatz zur Literatur zu ^23^Na-MRT im Rahmen der postoperativen Nachsorge – ein anfänglich hohes Natriumsignal prognostisch günstig zu sein. Jedoch ist die Evidenz aufgrund der geringen Kohortengröße mit auswertbarer ^23^Na-MRT zu Studienbeginn begrenzt. Zukünftig könnte die ^23^Na-MRT für die Auswahl von Patienten für eine „Watchful-Waiting-Strategie“ hilfreich sein.

## Introduction

Gliomas are the most common type of primary brain tumors. The 2021 World Health Organization (WHO) classification of brain tumors [1] refined the concept of a layered diagnosis of brain tumors based on histological, immunological, and genetic information. Prognosis is still correlated with the WHO grade, and diffuse low-grade gliomas (LGG) may be followed up using a “watchful waiting” concept, mainly if surgical resection is regarded high risk [2]. While recent data suggest that surgery and radio-/chemotherapy should no longer be reserved for progressive or anaplastic LGG, and that patients with diffuse LGG benefit from early surgical resection [3], some tumors are still regarded not resectable or not completely resectable, depending on their location. After an initial watchful waiting strategy, molecular tumor status was found to be the strongest determinant of progressive disease (PD; [4]). Histological grading of gliomas is based on the most malignant part of the tumor, and the degree of malignancy correlates with the extent of contrast enhancement, perfusion parameters, and spectroscopy results of ^1^H magnetic resonance imaging (MRI; [5]). In non-enhancing LGG, the most malignant part may be identified by dynamic susceptibility contrast perfusion MRI (DSC-MRI; [5]). ^23^Na-MRI is an interesting tool in clinical research [6, 7]. In contrast to conventional ^1^H MRI, ^23^Na-MRI uses the signal of tissue sodium, and image contrast expresses tissue sodium content, which differs between normal brain tissue and different histological subtypes of brain tumors [8]. We previously [5] reported excellent correlation between ^23^Na-MRI and DSC-MRI for the detection of the most anaplastic region in gliomas.

The aim of the present study was to evaluate whether ^23^Na-MRI can predict and/or, similarly to DSC-MRI, identify PD in patients with histologically proven LGG under a watchful waiting strategy.

## Materials and methods

### Study cohort

The study was performed according to the Declaration of Helsinki in its present form. Ethical approval was given by the local review board. Written and oral informed consent was obtained from all participants prior to inclusion in the study. Inclusion criteria were patients aged 18–80 years with newly diagnosed diffuse WHO grade II astrocytoma. Patients had to be treatment-naïve, the diagnosis of WHO grade II astrocytoma had to be proven histologically following stereotactic biopsy, and the chosen treatment strategy had to be active surveillance rather than primary resection. Exclusion criteria were any prior treatment of a brain lesion, any treatment strategy other than active surveillance, insufficient quality of MRI, lack of informed consent, and unwillingness to attend follow-up examinations at the participating institutions. During a 63-month period at two tertiary care centers in Germany, all patients meeting the criteria were prospectively and consecutively included.

All patients underwent stereotactic guided biopsy for histological evaluation. The biopsy site was identified as previously described [5]. All specimens were evaluated by two senior neuropathologists independently, blinded for the proposed WHO grade by the other reader. In cases of discordance, final histological grade was determined by consensus. After inclusion in the study, the patients underwent follow-up MRI examinations alongside their clinical visits. Visits were initially scheduled at 3‑month intervals, but the intervals varied greatly both intra- and inter-individually, ranging from less than 1 month to 12 months, with a mean interval of 4.9 months. For all patients undergoing ^23^Na-MRI at baseline, the intervals were 3 months or longer. Follow-up was ceased when imaging demonstrated PD according to the Response Assessment in Neuro-Oncology (RANO) criteria [9].

### MR imaging protocol

All MRI examinations were performed on a 3‑T whole-body scanner (Magnetom TIM Trio, Siemens Healthineers, Erlangen, Germany). For ^1^H imaging, a standard 32-channel head coil was used. For ^23^Na-MRI, images were acquired using a double-resonant (^1^H/^23^Na) quadrature birdcage head coil (Rapid Biomed, Rimpar, Germany) with three 0.3% NaCl solution phantoms placed beside the head of the patient within the coil. Structural MRI and perfusion MRI were performed during one session, immediately followed by the ^23^Na-MRI protocol after exchanging the head coils. The complete MRI protocol comprised the following sequences in chronological order: axial T2-weighted turbo spin echo (TSE), coronal T2-weighted TSE, axial T1 Flash 3D, chemical shift imaging spectroscopy (data not shown), during administration of a single dose of gadobenate dimeglumine (Gd-BOPTA, MultiHance®, Bracco Imaging, Milan, Italy; 0.1 mg/kg body weight, at an injection rate of 5 mL/s), echo planar perfusion-weighted imaging, followed by axial diffusion-weighted imaging, sagittal T2 SPACE FLAIR, axial T1 Flash 3D, and coronal T1 Flash 2D. DSC-MRI was performed using a T2*-weighted gradient-echo echo planar sequence (TR/TE 1440/47 ms, voxel size 1.9 × 1.9 × 5 mm^3^). ^23^Na-MRI was performed using a 3D radial gradient echo projection imaging sequence (TR/TE 60/0.2 ms; flip angle α = 82°; voxel size 4 × 4 × 4 mm^3^; 7500 projections; 2 averages; total acquisition time: T_acq_ = 15 min; [10, 11]).

### Image analysis

The MRI studies were pseudonymized and presented in a randomized order. All examinations were reviewed in consensus by two experienced board certified neuroradiologists blinded for clinical information. Tumor dimensions were evaluated in FLAIR, T2w, and CE-T1w images according to RANO criteria [9], using a PACS workstation (Centricity PACS, GE Healthcare). Progressive disease was defined as an at least 25% increase of the product of maximum diameter times the orthogonal diameter of the T2-hyperintense lesion, appearance of new lesions, or new contrast enhancement.

The DSC-MRI data were transferred to a dedicated workstation (NordicICE, Nordic Imaging Labs, Bergen, Norway) for further analysis [10]. Maximum relative regional cerebral blood flow (rrCBF) and relative regional cerebral blood volume (rrCBV) of the tumor were determined by using contralateral gray matter and white matter as internal reference.

The ^23^Na-MRI data were evaluated as described previously [5]. Image reconstruction was performed offline with Matlab (Mathworks, Natick, MA, USA). A Kaiser–Bessel gridding kernel was used followed by Hanning filtering and a conventional fast Fourier transform. Signal intensities were calculated using linear extrapolation. For tumor assessment, regions of interest (ROIs) of at least 20 pixels were placed on ^23^Na maps within the tumor tissue with maximum signal intensity (^23^Na_max_) as well as on a representative non-enhancing T2-hyperintense tumor area (^23^Na_tumor_) using the ^1^H MR images as reference for orientation. The ROIs were placed in consensus with an experienced MRI physicist, familiar with the ^23^Na-MRI sequence, to recognize sequence specific image artifacts and possible B1-field non-uniformity between the different coils, which might interfere with ROI analysis. For interindividual comparisons, the signals obtained from tumors were normalized on sodium signals of different extrinsic and intrinsic references. The values of the ROIs placed on the tumor were divided by the mean values of the three phantoms (^23^Na_tumor/phantom_ and ^23^Na_max/phantom_), healthy white matter (^23^Na_tumor/wm_ and ^23^Na_max/wm_), or vitreous humor (^23^Na_tumor/vh_ and ^23^Na_max/vh_). To prevent a reading bias, the imaging analysis of perfusion MRI and ^23^Na-MRI was performed on separate days.

### Statistical analysis

Statistical analysis was performed by an independent statistician using the statistical software R (version 2.10.1; The R Foundation for Statistical Computing, Vienna, Austria) with R package survival (version 2.35-7) and R package coxphf (version 1.05). The primary endpoint of the study was time to progression (TTP), defined as time from the initial visit (baseline) to the detection of PD by means of MRI, which was analyzed using the Kaplan–Meier method. Patients without PD were censored at the last follow-up examination. To identify the prognostic impact of baseline values of DSC-MRI and ^23^Na-MRI in only one group with identical diagnosis and without treatment subgroups, univariate Cox proportional hazard regression modeling was carried out. Multivariate Cox regression analysis was not possible due to the small sample size of patients undergoing baseline ^23^Na-MRI. In all statistical tests, an effect was considered statistically significant if the *P* value was 0.05 or less. *P* values were not adjusted for multiple testing and interpretation of *P* values was explorative.

## Results

During the study period, 30 patients underwent baseline MRI for suspected LGG. Of these patients, 12 (40%) had to be excluded: seven patients decided against further visits at the participating centers and were therefore lost to follow-up after baseline imaging; in five patients, the histological diagnosis was not WHO grade II astrocytoma (one WHO grade I astrocytoma, one WHO grade III astrocytoma, one anaplastic astrocytoma, and in two cases no biopsy could be carried out), and evaluation of MR datasets was not possible due to artifacts in one patient. Thus, the study population consisted of 18 patients with newly diagnosed, biopsy-proven, and treatment-naïve WHO grade II astrocytoma (10 women, 8 men; age at the time of baseline imaging, 42 **±** 15 years [median 45 years; range 19–66 years]). The main results are summarized in Tables 1 and 2.

**Table 1:** Characteristics of the six patients with baseline ^23^Na-MRI. Summary of individual DSC-MRI and ^23^Na-MRI parameters at baseline and at PD

ID	Age	TTP	rrCBV BL	rrCBF BL	rrCBV PD	rrCBF PD	^23^Na_tumour/phantom_ BL	^23^Na_max/phantom_ BL	^23^Na_tumour/phantom_ PD	^23^Na_max/phantom_ PD
4	20	98	1.75	1.79	1.56	1.63	1.34	1.39	0.97	0.97
11	20	558	1.54	1.69	1.75	1.87	2.29	2.36	2.53	2.68
19	66	633	1.32	1.24	1.20	1.23	1.79	2.02	1.93	2.25
21	21	371	1.81	1.81	n. a.	n. a.	1.61	1.77	3.67	4.14
24	51	297	1.83	1.92	1.82	1.94	1.86	1.87	1.68	1.74
26	61	92	1.86	1.97	n. a.	n. a.	1.18	1.43	n. a.	n. a.

*ID* random numerical pseudonyms, *TTP* time to progression, *rrCBV* regional relative cerebral blood volume, *rrCBF* regional relative cerebral blood flow, *BL* baseline, *PD* time point at diagnosis of progressive disease, *n.* *a.* not available

**Table 2:** Results of perfusion and ^23^Na-MRI analysis at baseline. Summary of DSC-MRI and ^23^Na-MRI parameters at baseline and HRs on endpoint of time to progression^a^

	*n*	Mean	Median	min	max	HR	95% CI
Age	18	42.9	44.8	19.7	66.5	–	–
rrCBV	17	1.6	1.6	0.8	3.0	1.39	0.88–2.19
rrCBF	17	1.7	1.7	0.9	3.1	1.29	0.81–2.06
^23^Na_tumor/phantom_	6	2.0	1.8	1.2	3.7	0.90	0.32–2.59
^23^Na_max/phantom_	6	2.2	1.9	1.4	4.0	0.91	0.34–2.45
^23^Na_tumor/wm_	6	1.5	1.5	0.9	1.9	0.86	0.56–1.31
^23^Na_max/wm_	6	1.6	1.7	0.9	2.1	0.75	0.26–2.18
^23^Na_tumor/vh_	6	0.7	0.7	0.5	0.8	0.82	0.25–2.69
^23^Na_max/vh_	6	0.8	0.8	0.5	0.9	0.91	0.39–2.09
^23^Na_tumor/bn_	6	19.7	18.3	14.7	27.3	0.24	0.02–2.36
^23^Na_max/bn_	6	21.3	19.3	15.5	29.5	0.34	0.05–2.39

Sodium contents in representative non-enhancing T2w-hyperintense parts of the tumors (^23^Na_tumor_) and tumor areas with maximum sodium values (^23^Na_max_) normalized to mean values of three sodium phantoms (^23^Na_tumor/phantom_ & ^23^Na_max/phantom_), healthy white matter (^23^Na_tumor/wm_ & ^23^Na_max/wm_), vitreous humor (^23^Na_tumor/vh_ & ^23^Na_max/vh_), and background noise (^23^Na_tumour/bn_ & ^23^Na_max/bn_)

*CI *confidence interval, *HR* hazard ratio,* rrCBF* regional relative cerebral blood flow

*P* > 0.05 for all values

^a^Derived by univariate Cox regression analysis

Morphological MRI was performed for all patients. According to the RANO criteria, PD was found in 14 patients (78%) after a mean interval of 420 ± 354 days (median: 290 days; range: 92–1154 days).

**Fig. 1:**
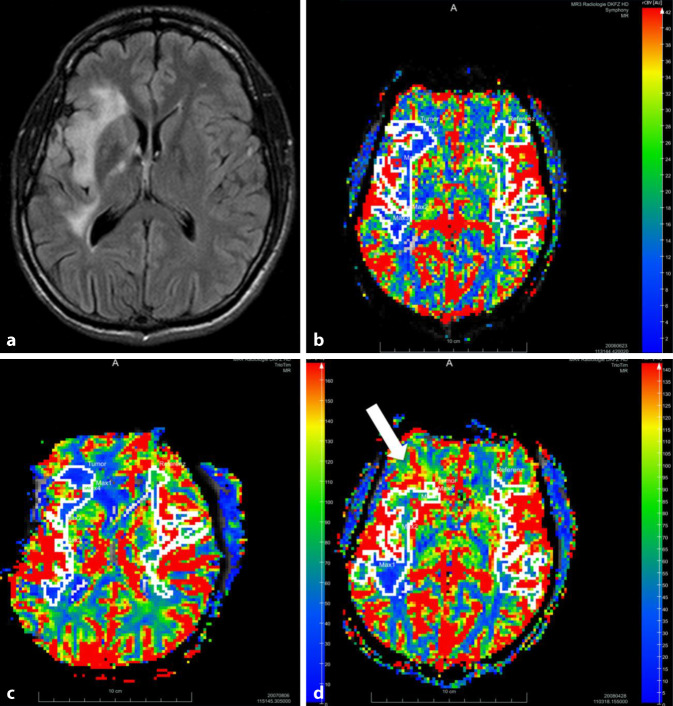
Surveillance with perfusion MRI. A 37-year-old patient with WHO grade II astrocytoma in the right hemisphere visualized on representative axial FLAIR (**a**) image at baseline. DSC-rrCBV maps at baseline (**b**) and at the 14-month follow-up (**c**) do not show elevated rrCBV values in the tumor region. At the 20-month follow-up (**d**), the time point of progressive disease as determined by RANO criteria, rrCBV elevation is seen in the right frontal and insular subcortical white matter (white arrow)

**Fig. 2:**
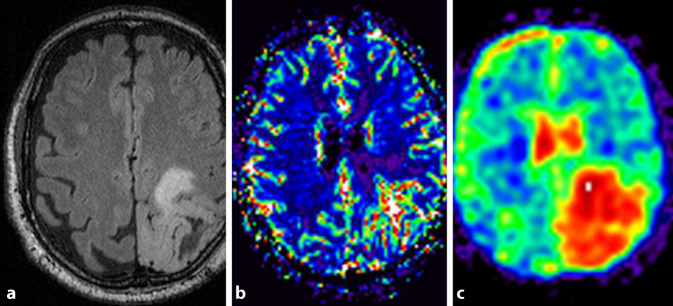
Multiparametric MRI protocol including ^23^NA-MRI. A 59-year-old patient with WHO grade II astrocytoma in the left hemisphere with multimodal MRI including DSC-MRI and ^23^NA-MRI at baseline. **a** Representative axial FLAIR image. At baseline, rrCBV in the tumor was elevated 1.66-fold compared to healthy contralateral white matter (**b**). Corresponding axial ^23^NA-MRI slice (**c**) as color-coded parameter map. Relative sodium content within the tumor was 1.99 when compared to contralateral healthy white matter at baseline. At the 22-month follow-up, the time point of progressive disease as determined by RANO criteria, relative sodium content decreased to 1.77 (not shown)

Magnetic resonance perfusion imaging was performed in 17 patients. An exemplary evaluation of DSC perfusion is shown in Fig. 1. For rrCBV, the hazard ratio (HR) was 1.39 (95% CI 0.88–2.19). For rrCBF, the result was an HR of 1.29 (95% CI 0.81–2.06). The *P* values were > 0.05 for both parameters. Not all patients were able to undergo ^23^Na-MRI, owing to either individual issues impeding the ability to add more than 15 min of scanning time to the already lengthy multimodal brain tumor MRI protocol, or due to missing capacities at the dedicated MRI scanner compatible with the ^23^Na-coil. ^23^Na-MRI could be performed on ten patients. However, in three patients adequate sodium imaging was missing at baseline, and in one patient the sodium phantoms were missing; thus, only six datasets were available for evaluation at baseline (Table 1). All six patients with baseline ^23^Na-MRI experienced PD. In two patients (no. 4 and no. 26), PD was diagnosed as early as during the 3‑month follow-up (92 and 98 days, respectively). Patients 11 and 19 had follow-up examinations in 3‑month intervals regularly until PD was diagnosed after 558 days and 633 days, respectively. Patient 21 had one 6‑month interval between the baseline and the first follow-up examination, followed by two regular 3‑month intervals until PD after 371 days. Patient 24 had one 6‑month interval between the baseline and the first follow-up examination as well, followed by a 4-month interval until PD after 297 days. The individual imaging results for these six individuals were inhomogeneous. Patient 4 had an rrCBV of 1.75 and a ^23^Na_tumor/phantom_ value of 1.34 at baseline. When PD was diagnosed 98 days later, rrCBV was 1.56 and ^23^Na_tumor/phantom_ had dropped to 0.97. In patient 11, rrCBV was 1.54 and ^23^Na_tumor/phantom_ was 2.20 at baseline, and both values had increased to 1.75 and 2.53, respectively, at the time point of PD 558 days later. At baseline, patient 19 had an rrCBV of 1.32 and a ^23^Na_tumor/phantom_ value of 1.79; rrCBV decreased slightly to 1.20, whereas ^23^Na_tumor/phantom_ increased to 1.93 at PD after 633 days. In patient 21 we found a baseline rrCBV of 1.81, but no DSC perfusion data were available at the timepoint of PD 371 days later; baseline ^23^Na_tumor/phantom_ was 1.61 and rose to 3.67. In patient 24, rrCBV remained nearly unchanged from baseline to PD after 297 days (1.82 to 1.83), but ^23^Na_tumor/phantom_ values dropped from 1.86 to 1.68. In patient 26—the patient with the fastest PD in our cohort after only 92 days—neither perfusion MRI nor ^23^Na-MRI data were available at PD; baseline values were rrCBV 1.86 and ^23^Na_tumor/phantom_ 1.18. An exemplary evaluation of a ^23^Na-MR study is shown in Fig. 2. In summary, when normalized to the mean sodium signals of the phantoms, HRs were 0.90 (95% CI 0.32–2.59) for ^23^Na_tumor/phantom_ and 0.91 (95% CI 0.34–2.45) for ^23^Na_max/phantom_, respectively. When normalized to intrinsic references, i.e., healthy white matter and vitreous humor, there was a tendency for an even lower HR. The *P* values were > 0.05 for all parameters.

## Discussion

Here, we present the initial findings on ^23^Na-MRI as an addendum to multimodal MRI in histologically proven LGG under a “watchful waiting” strategy and describe the possible added diagnostic value of this imaging biomarker.

The ^23^Na MR signal in vivo is 22,000 times smaller than that of ^1^H and decays bi-exponentially. With dedicated radiofrequency coils and imaging sequences with ultra-short echo times of less than 1 ms, it is possible to observe tissue sodium signal as a weighted average of intracellular [^23^Na]_i_ and extracellular [^23^Na]_e_ sodium [8], with complex relations in healthy tissues and even more so in tumors [12]. Changes in [^23^Na]_i_ are linked to cell proliferation and cell integrity. Therefore, ^23^Na-MRI proved to be a promising tool for investigating brain tumors in various experimental studies [13, 14]. Although ^23^Na-MRI is increasingly implemented, applications are currently limited to clinical research [6, 7]. Reasons for this include the need for costly dedicated coils, extended measurement times, complex data postprocessing, and relatively low spatial resolution [14]. Many studies suffer from small sample sizes and methodological heterogeneity, ranging from different field strengths (1.5 to 7.0 T) over a variety of tumor entities in one publication, heterogenous imaging protocols, and different treatment strategies studied to different parameters derived from the ^23^Na-MRI data [6, 13]. While normal values of tissue sodium content have been described, specific reliable and clinically applicable cutoff values for pathology are not established [12].

Our finding that substantial elevation of rrCBV and rrCBF in the tumor favors PD in WHO grade II astrocytoma is in line with the literature and underlines the high value of DSC-MRI as a standard in brain tumor MRI protocols [15].

High sodium content in the tumor at baseline showed a tendency to being prognostically favorable in our small cohort of patients with WHO grade II astrocytomas under a watchful waiting strategy who underwent ^23^Na-MRI. Of the six cases with available baseline ^23^Na-MRI, the two individuals with the highest maximum baseline ^23^Na values experienced the longest time to progression and in both cases the values further increased at disease progression. On the contrary, the two patients with the lowest initial ^23^Na values experienced the shortest time to progression. In one of these two patients, ^23^Na-MRI data were available at the time point of disease progression and ^23^Na values had interestingly dropped further. In the two patients with intermediate TTP, ^23^Na values were also between the other pairs’; however, in one individual the values rose at the time point of disease progression, while in the other they fell. By contrast, our group previously showed a positive correlation between elevated sodium signal and functional ^1^H imaging parameters (rCBV and chemical shift imaging) in targeting the most anaplastic tumor region for biopsy with a comparable technique; however, this was done at 1.5 T [5]. Using a more advanced ^23^Na-MRI sequence, allowing for quantification of tissue sodium concentration (TSC), Biller et al. were able to demonstrate a positive correlation between a TSC ratio and IDH mutation status as well as tumor progression in a variety of brain tumors of WHO grades I–IV [13]. High TSC is regarded a biomarker for cell pathology because [^23^Na]_i_ increases in cases of leaky cell membranes or impaired energy metabolism [8], as well as in proliferating and immature cells [12]; the higher the value, the more dysplastic the tumor cells, thus linking it to IDH mutation status and WHO grades [13, 16]. Leon-Benedetti et al. recently reported the case of a WHO grade III astrocytoma with clinical concern for progression after resection, where a sustained decrease in total sodium concentration contributed to the correct diagnosis of pseudo-progression rather than true progression [17]. However, contrary to these studies, we focused on differences in a specific homogeneous subgroup of biopsy-proven, treatment-naïve WHO grade II astrocytomas only, under a watchful waiting strategy. Homogeneity of the histological tumor entities was aimed for by biopsy targeting through multimodal MRI, reducing the risk of sampling errors. The cohort of patients undergoing ^23^Na-MRI at baseline was also relatively homogeneous with regard to follow-up intervals, as in the majority regular 3‑month intervals were realized, with two exceptions of longer 6‑month intervals between baseline and first follow-up. Interestingly, in terms of sodium signals, this group was not as homogeneous. Moreover, our results yielded a caveat for interpreting changes of the sodium signals during follow-up examinations, as we found both increasing and decreasing signals at the time point of PD in different patients. We would like to add that in one individual that we could not include into the evaluation because of missing baseline ^23^Na-MRI, we detected high relative sodium content in the tumor throughout ten follow-up examinations (data not shown), and the patient did not experience PD during the study period. While stable ^23^Na values in follow-up examinations might, in theory, seem a possible indicator of stable disease, this one case—with missing baseline data on top of that—does not adequately support this assumption. Unfortunately, we do not have additional genetic information on the astrocytomas, such as the IDH mutation status [18]. The reasons for the differences in sodium content at baseline and in the follow-ups are probably to be found in the complex environment the tumor exerts. On the one hand, higher sodium levels may reflect cell proliferation, cell dysplasia, and cell damage, as described—all of which are factors that we would interpret as unfavorable in brain tumors. On the other hand, sodium content may also be elevated by changes in the extracellular space, e.g., through extracellular space expansion by edema. In malignant brain tumors, including high-grade glioma, peritumoral edema is thought to be mainly caused by blood–brain barrier disruption and tumor angiogenesis, and therefore is an unfavorable prognostic factor, with the added risk of acute deterioration through elevated intracranial pressure and brain herniation [19]. However, edema formation is neither completely understood nor regarded a dominant feature of LGGs [19, 20]. In the evaluation of sodium content, different ROIs were placed within the tumor margins in the region of the maximum sodium signal as well as in a representative non-enhancing area; hence, signal contamination through peri-tumoral edema was unlikely. Intra-individual differences in normalized sodium signals of the different tumor regions were relatively small regardless of T2-inhomogenities and did not raise suspicion of pronounced heterogeneity in the tumor matrix, e.g., caused by assumed intra-tumoral edema. Further, extracellular sodium content may also be altered by changes in molecular and proteinaceous components of the extracellular matrix that are not necessarily linked to tumor aggressiveness [12, 19]. As the ^23^Na-MRI technique used in this study did not allow for differentiation of intra- and extracellular sodium content, we cannot further elucidate differences in the composition of tumor environments that may have affected prognosis.

### Limitations

The main limitation of our study lies in the small sample size, especially in patients undergoing ^23^Na-MRI, albeit not addressing a rare tumor entity. However, we wanted to focus on WHO grade II astrocytomas under a watchful waiting strategy specifically, which limited the number of eligible candidates. Nonetheless, we were able to provide uniform morphological baseline imaging and histopathologic workup. Other problems were limitations in the ^23^Na-MRI sequence used that could not readily differentiate intra- and extracellular components of sodium and only allowed for semiquantitative evaluation of sodium levels, as well as the missing IDH status. Thus, the determination of cutoff values that would allow for individual risk prediction was not feasible.

## Conclusion

In monitoring primary non-resectable WHO grade II astrocytoma in a “watchful waiting” concept, the parameters relative regional cerebral blood volume and relative regional cerebral blood flow in the tumor predict disease progression. It is possible to carry out ^23^Na-MRI for WHO grade II astrocytoma surveillance. An initially high sodium signal in the tumor might be prognostically favorable in some cases. Careful interpretation of changing sodium signals is necessary in follow-up examinations, because in our cohort progression was encountered with both increasing and decreasing values. Future studies on the topic will profit from advances in the ^23^Na-MRI technique as well as from neuropathology and should include more patients prospectively, when applicable, under a “watchful waiting” strategy.

## Highlights

- Dynamic susceptibility contrast perfusion (DSC)-MRI has become a well-established biomarker for tumor grading, guiding stereotactic biopsy and detecting disease progression in brain tumors.
- ^23^Na-MRI is an interesting addendum to brain tumor MRI protocols in clinical research.
- ^23^Na-MRI represents a promising imaging biomarker with potential as a prognostic factor at baseline, helping to define patients with WHO grade II astrocytomas that benefit from “watchful waiting” rather than early resection.

## Data Availability

The data that support the findings of this study are not openly available due to reasons of sensitivity and are available from the corresponding author upon reasonable request.

## Declarations

All procedures performed in studies involving human participants or on human tissue were in accordance with the ethical standards of the institutional and/or national research committee and with the 1975 Helsinki declaration and its later amendments or comparable ethical standards. Ethical approval was given by the Ethics Committee of the Medical Faculty of Heidelberg University. Informed consent was obtained from all individual participants included in the study.

The supplement containing this article is not sponsored by industry.
